# The Possibility of Using Components of Plant Origin as Reinforcements in Composite Friction Materials—A Simulation-Based Braking Process Study

**DOI:** 10.3390/ma17235834

**Published:** 2024-11-28

**Authors:** Andrzej Borawski, Dariusz Szpica, Grzegorz Mieczkowski

**Affiliations:** Faculty of Mechanical Engineering, Bialystok University of Technology, 45C Wiejska Str., 15-351 Bialystok, Poland; d.szpica@pb.edu.pl (D.S.); g.mieczkowski@pb.edu.pl (G.M.)

**Keywords:** friction, wear, heating, brakes, simulation

## Abstract

An innovative prototype composition of a composite friction material was developed. The actual values of selected parameters were determined, as described in a previous paper. It was decided to verify whether the proposed material differs from conventional materials in terms of temperature characteristics, and if so, to what extent. For this purpose, numerical studies were performed using the problem of initially boundary thermal conductivity. The braking system of a popular passenger car was used as the object of the research. A mathematical model of the studied phenomenon was developed, which was implemented in a virtual environment. The results showed that changing the reinforcement method to a more ecological one than the conventional one does not cause significant changes in the temperature profiles obtained for the adopted braking scenario.

## 1. Introduction

The number of vehicles used by people is constantly growing. The openness of the market means that people find employment in companies located even several hundred kilometers away from their place of residence, and the availability of products is widespread, with products being available almost instantly. These are very big advantages from the point of view of consumers, unfortunately they are a logistical challenge related to the need for frequent, long-ranging, and fast transport [1,2].

This idea is unfortunately contrary to the principles of ecology. A large number of vehicles translates into large amounts of pollutants emitted into the atmosphere. These pollutants include both gases from exhaust systems and solid particles that are products of the abrasive wear of tires and the working elements of brake systems [3,4]. To address these factors, very restrictive regulations are being introduced, drastically reducing the permissible level of pollutants for vehicles [5,6].

Vehicle brakes are types of energy converters. They convert the kinetic energy of movement into thermal energy. This energy, in turn, is released into the immediate environment—the atmosphere and suspension elements [7,8,9]. The quality of brake operation depends on effective and rapid heat release, because exceeding the critical temperature value can lead to the loss of material cohesion and, thus, the loss of braking ability. This phenomenon is called fading [10,11,12,13].

Properties, including mechanical, tribological, and thermal properties, largely depend on the composition of the composite friction material. In such a material, we can find four groups of components. According to the function they perform, they can be divided into the following categories: friction modifiers, fillers, matrices, and reinforcements. These can be materials of both natural and synthetic origin, with materials of the latter being more harmful and those of the former being less harmful [14,15,16].

One of the most important roles is played by reinforcement materials [17,18]. Before the introduction of ecological regulations, the most commonly used fibrous reinforcement material was asbestos. Its temperature resistance and good mechanical properties meant that manufacturers were extremely eager to use it. Unfortunately, it turned out that asbestos has strong carcinogenic properties. Shortly after this discovery, it was completely withdrawn from use [19,20,21].

Today’s level of technological development allows for the production of a wide variety of materials aimed at achieving the desired properties. As shown in [22], the type of reinforcement used significantly affects the mechanical properties of the material. Currently, aramid or carbon fiber is most often used for reinforcement. Unfortunately, both of them generate serious pollution. Aramid is produced in the low-temperature polycondensation process of para-phenylenediamine (PPD) and terephthaloyl chloride (TCL) monomers. PPD is a highly sensitizing aromatic amine. In industry, it is used for dyeing hair, fur, and fabrics. The by-product of its fabrication is hydrochloric acid. The production of aramid fibers and fabrics is expensive due to the difficulty of using the concentrated sulfuric acid needed to maintain the water-insoluble polymer during synthesis and spinning [23,24]. Carbon fiber is also a harmful material. Its production process produces very fine dust. Its small size means that it can easily get into hard-to-reach places of machines and devices, which can cause damage. In the case of contact with humans, it can cause inflammation of the skin or mucous membranes. For this reason, it is necessary to use uncomfortable protective clothing [25,26]. Replacing these materials with something more ecological is therefore an important scientific goal.

The quality of composite friction materials can be tested in many ways [27]. Depending on the test object used, the following can be conducted [28,29]:

- Road tests, in which a real vehicle and its natural operating conditions are used;
- Bench tests, in which an entire vehicle is used but in fixed conditions prepared by the person performing the test;
- Component testing that features the use of the entire, complete component, which allows for the interactions of individual parts and their impact on the results to be taken into account;
- Component testing in which a disk or pad is used and testing is carried out in fixed laboratory conditions;
- Theoretical testing, which is carried out using a mathematical model of the phenomenon being tested and, most often, a computer with FEM software (COMSOL Multiphysics, https://www.comsol.com/comsol-multiphysics).

Simulation tests are most often performed. They allow for the representation of very complicated operating conditions, with such tests having a low total cost. However, it is necessary to assume certain simplifying conditions. In order to reduce the resulting errors, the method described by Stevens et al. [30] can be used, which uses first-order differential equations. You et al. used different methods for different materials with different microstructures [31]. Esfe et al. showed that, in addition to the composition of the material itself, which is the subject of our research, the course of friction heating is also influenced by the Reynolds number or the size of the particles used in production [32]. Similar studies were conducted by Kumar et al. [33]. They revealed the relationship between the Reynolds number and convective heat flow. It also turns out that there is a relationship between Nusselt number values, Reynolds number, and the disk-to-wheel-diameter ratio, which was investigated by Zhang et al. [34]. The correct values allowed for more effective heat transfer by conduction. Moraveji et al. showed, in turn, that the time profile of the temperature is also influenced by the vortex tube, an increase in the number of which contributes to the lowering of the output temperature [35]. Ghandouri et al., in their studies, checked the influence of geometrical features on the brake heating process. In their simulations, they used new types of ribs, which allowed for an improvement in the heat transfer coefficients [36]. Song et al. also made holes in the ribs. They demonstrated a positive effect on the heat release rate [37]. The proposed composite materials intended for braking systems can help protect the environment, as harmful components are eliminated from their composition. It was proven that replacing aramid with flax fibers does not negatively affect the tribological properties. The aim of the research described in this paper was to check whether the modification introduced in the reinforcement affects the course of friction heating in the real braking system, and if so, to what extent. For this purpose, simulation studies were used, and they were carried out in the Comsol environment. An initial-boundary problem was developed, taking into account friction heating. Thermal contact was assumed to be perfect.

## 2. Materials and Methods

For the purpose of the research, four groups of composite friction material samples dedicated to vehicle braking systems were prepared. The input variable, allowing for the differentiation of the tests and achieving the goal assumed in the introduction, was the change in the composite reinforcement of the friction material. In sample S1, an aramid reinforcement was used—a type of reinforcement used very often in such materials. In the subsequent samples (S2–S4), the aramid content was reduced until it was completely removed. Flax fibers were introduced in place of the aramids. Their content was gradually increased from 4% by weight in sample S2 to 12% in sample S4. Details regarding the composition of samples are presented in Table 1.

The components of each group were measured using a Steinberg SBS-LW-300A laboratory scale (accuracy 10^−3^ g, Hamburg, Germany). The material was then placed in the mixing device described in [38]. Mixing was performed for one hour. The speed was regulated using a stepper motor and a control module based on an Arduino board. After mixing, the samples were placed in molds and subjected to a pressure of 20 MPa. Then, they were heated at 60 °C for 24 h. The finished samples were subjected to abrasive processing in order to obtain the desired geometry, dimensions, and surface quality. Then, the samples were transferred to an external company to determine the thermo-mechanical properties necessary for the simulation tests. The necessary data for the brake disk made of gray cast iron were selected from the manufacturer’s data. The obtained data are presented in Table 2.

**Figure 1:**
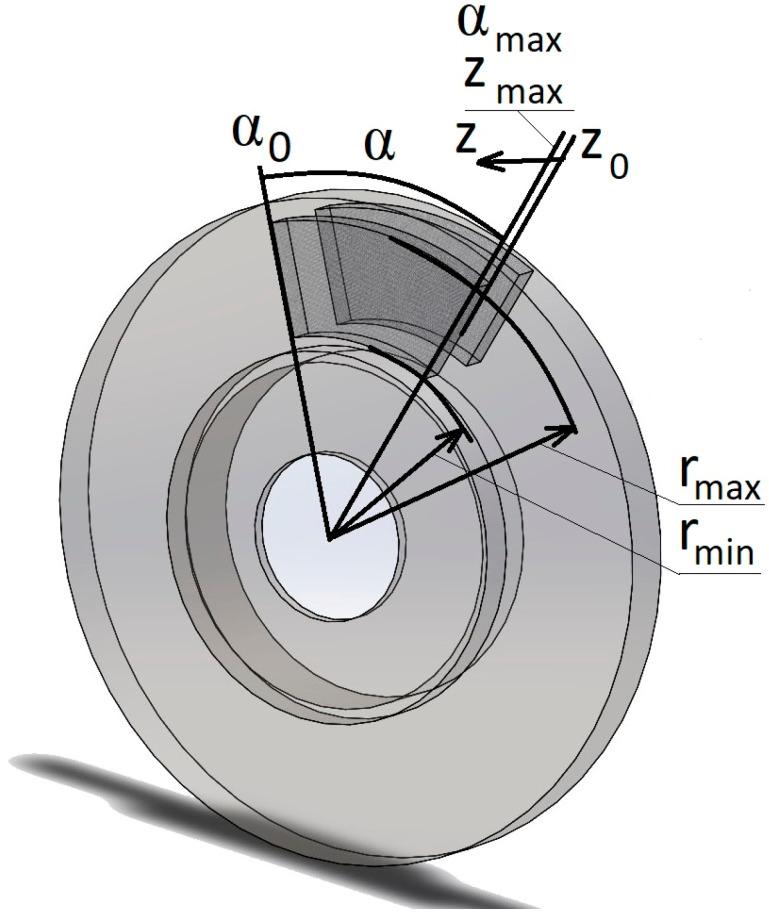
Three-dimensional model prepared for numerical calculations.

The disk brake system of a popular passenger car was selected as the object of the research. Information on the necessary data, i.e., vehicle mass and geometry of working brake elements, was taken from catalog data. The 3D model was created in the SolidWorks environment and then implemented into Comsol Multiphysics software (https://www.comsol.com/comsol-multiphysics). In further considerations, the brakes of one front wheel were taken into account. The calculation scheme is shown in Figure 1.

The research carried out as part of this work was comparative in nature. The comparison consisted of carrying out tests of a material whose composition is similar to commercial brake pads and then carrying out the same tests, in the same conditions, using a developed composite. Such testing will allow you to determine whether the change introduced will affect the heating process during braking without the need for expensive production of the actual braking system and its testing.

Due to the fact that virtual tests have limited possibilities, it was necessary to adopt certain simplifying assumptions. They did not significantly affect the final result but did help significantly in facilitating the mathematical description of the braking process under consideration. These assumptions were as follows:

- The pad material is homogeneous, while in reality, it consists of individual components.
- External factors such as gusts of wind or road unevenness do not affect the braking process, while in reality, they improve or impede the effectiveness of the brakes.
- The coefficient of friction between the disk and the pad does not show temperature sensitivity, while in reality, its value changes.
- Braking occurs without slippage of the vehicle wheels, with a constant deceleration equal to the acceleration of gravity.
- Friction occurs over the entire surface of the pad, and the contact surface is perfectly flat, while in reality, it has depressions and irregularities.
- The ambient temperature is constant and equal to 25 °C.
- The contact pressure increases and decreases without delay.

It was assumed that the kinetic energy of motion is entirely converted into thermal energy as a result of friction. Hence, the energy absorbed by the braking system is as follows:

(1) $$E_{kc}(t)=\frac{m_{v}\cdot v^{2}}{2},\;t_{1}\leq t\leq t_{2},$$

where *t*_1_—brake engagement time, *t*_2_—vehicle stopping time, *m_v_*—vehicle mass, *t*—time, and *v*—initial vehicle speed. It was assumed that the vehicle will brake from an initial speed of 90 km/h (25 m/s). In a disk brake system, the braking torque can be written as follows:

(2) $$M_{f}\left(t\right)=fpAr_{c},\;t_{1}\leq t\leq t_{2},$$

where *f*—value of the friction coefficient for the sample (S1–S4) determined experimentally in the previous work [39], *p*—contact pressure, *A*—surface area of the brake pad, and *r_c_*—mean friction radius, which can be determined from the following formula:

(3) $$r_{c}=\frac{2\pi }{A}\int_{r_{min}}^{r_{max}}r^{2}dr=\frac{2(r_{max}^{3}-r_{min}^{3})}{3(r_{max}^{2}-r_{min}^{2})},\;r_{min}\leq r\leq r_{max},$$

and the contact surface area (of the pad) is as follows:

(4) $$A=2(\frac{\alpha_{max}}{\pi }(r_{max}-r_{min})),$$

where *r_min_*—the smallest contact radius of the pad with the disk, *r_max_*—the largest contact radius of the pad with the disk, and *α*—the angular sector of the disk in contact with the brake pad. After transformation, the contact pressure value takes the following form:

(5) $$p=\frac{M_{f}(t)}{f\cdot A{\cdot r}_{c}},\;t_{1}\leq t\leq t_{2}.$$

The friction moment can also be made dependent on the moment of inertia *I*; then, we obtain the following:

(6) $$M_{f}\left(t\right)=-I\frac{d\omega (t)}{dt}\;,\;t_{1}\leq t\leq t_{2},$$

where the moment of inertia is

(7) $$I=\frac{{2E}_{k}(t)}{{\omega (t)}^{2}r_{c}},\;t_{1}\leq t\leq t_{2},$$

The friction force required to achieve braking is

(8) $$P\left(t\right)=fpV(t),\;{\;t}_{1}\leq t\leq t_{2},$$

where the linear velocity of the pad in motion relative to the disk *V* at the central point is

(9) $$V\left(t\right)=\omega (t)\;r_{c},\;{\;t}_{1}\leq t\leq t_{2}.$$

In the case under consideration, it was assumed that each braking ends with a complete stop. It can therefore be written that the energy absorbed by the brakes is equal to the work that must be put in to stop the vehicle:

(10) $$E_{k}\left(t\right)=-∆W,\;{\;t}_{2}\leq t.$$

The temperature distribution in friction elements in cylindrical coordinates was obtained by calculating the initial-boundary thermal conductivity problem, taking into account the heating due to friction. According to Fourier’s law,

(11) $$\begin{array}{c} \frac{\partial^{2}T}{\partial r^{2}}+\frac{1}{r}\frac{\partial T}{\partial r}+\frac{1}{r^{2}}\frac{\partial^{2}T}{\partial \alpha^{2}}+\frac{\partial^{2}T}{\partial z^{2}}=\frac{1}{k_{d}}\left(\frac{\partial T}{\partial t}+\omega \frac{\partial T}{\partial \alpha }\right),\\ {\;t}_{1}\leq t\leq t_{2},\;{\;r}_{1}\leq r\leq r_{c},\;0\leq \alpha \leq \alpha_{max},\;0\leq z\leq z_{max}, \end{array}$$

where *k*—thermal conductivity, *ρ*—material density, and *c_p_*—specific heat. It was also assumed that both during braking and after the vehicle has come to a complete stop, cooling occurs due to conduction. This is expressed as follows:

(12) $$\dot{q}=-k\frac{T_{max}-T_{0}}{z_{max}},\;0\leq z\leq z_{max}.$$

According to the manufacturer, the vehicle’s ready-to-drive weight is 1950 kg. Taking into account the driver’s weight, the following can be assumed:

(13) $$m_{v}=2040\;kg.$$

The manufacturer also stated that the weight distribution is around 63 ÷ 37%. Therefore, the normal force on the front axle is as follows:

(14) $$F_{nf}=0.63\cdot 2040\;kg\cdot 9.81\frac{m}{s^{2}}\approx 12.6\;kN.$$

This gives a normal force on one wheel of

(15) $$F_{nfp}=F_{nfl}=\frac{12.6\;kN}{2}=6.3\;kN.$$

The calculations also assume that the vehicle’s center of gravity remains in the same place during braking.

## 3. Results and Discussion

**Figure 2:**
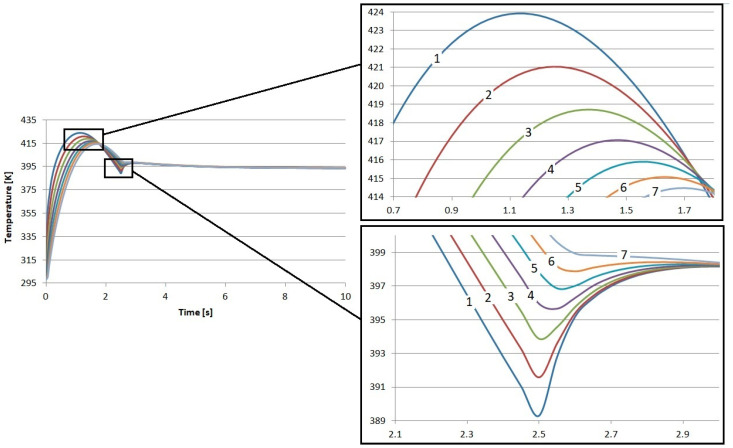
Numerical calculation results of material S1: 1—depth 0.05 mm, 2—depth 0.5 mm, 3—depth 1 mm, 4—depth 1.5 mm, 5—depth 2 mm, 6—depth 2.5 mm, and 7—depth 3 mm.

**Figure 3:**
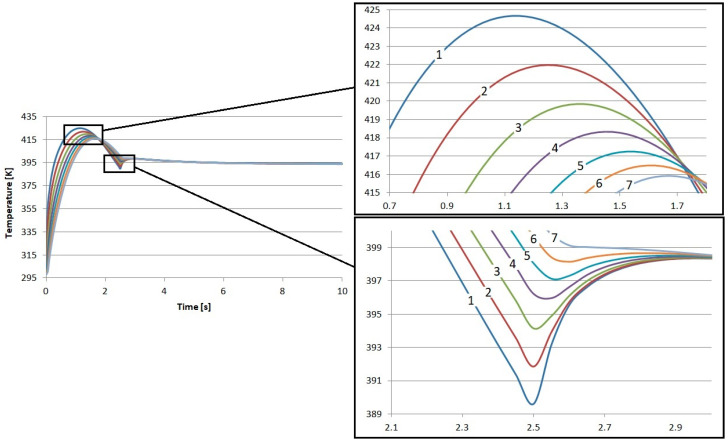
Numerical calculation results of material S2: 1—depth 0.05 mm, 2—depth 0.5 mm, 3—depth 1 mm, 4—depth 1.5 mm, 5—depth 2 mm, 6—depth 2.5 mm, and 7—depth 3 mm.

**Figure 4:**
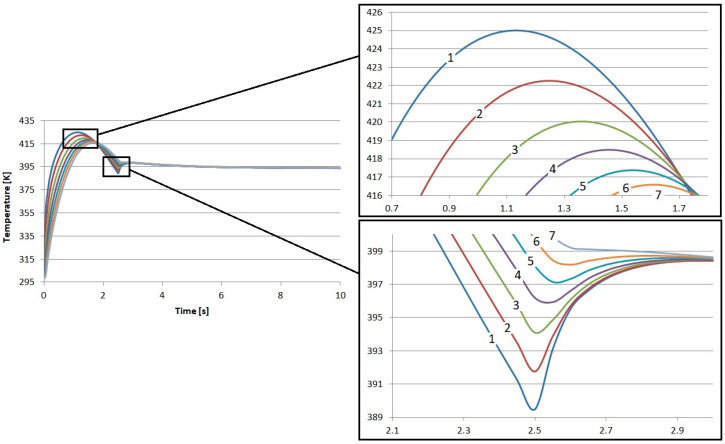
Numerical calculation results of material S3: 1—depth 0.05 mm, 2—depth 0.5 mm, 3—depth 1 mm, 4—depth 1.5 mm, 5—depth 2 mm, 6—depth 2.5 mm, and 7—depth 3 mm.

**Figure 5:**
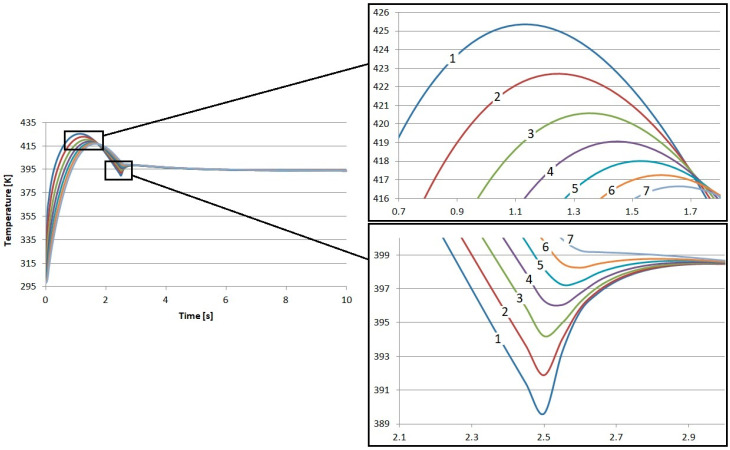
Numerical calculation results of material S4: 1—depth 0.05 mm, 2—depth 0.5 mm, 3—depth 1 mm, 4—depth 1.5 mm, 5—depth 2 mm, 6—depth 2.5 mm, and 7—depth 3 mm.

The direct results of the research were the temperature profiles of the friction heating process of the proposed composite friction materials. In each case, nearly 29 k degrees of freedom (plus 14.3 k internal DOFs) were taken into account during the simulation. In each case, the results reflect the time from the beginning of braking, through complete stopping, and immediately after braking. In total, the calculations were performed for 10 s. The results of the calculations regarding the temperature inside the S1–S4 materials are presented in Figure 2, Figure 3, Figure 4 and Figure 5. In each case, the measurements were taken at different depths: from the contact plane to a depth of 3 mm with a step of 0.5 mm. The exception was the first point, where the measurement depth was 0.05 mm.

The results obtained for the individual materials are very similar. The highest temperature value was recorded for material S4. It was 425.3 K, and this value was obtained after 1.15 s from the start of braking. The lowest maximum temperature was for material S1. Its value was 423.9 K, also obtained 1.15 s from the start of braking. It is worth noting that this difference is only less than 1.5 K. This is a value that will not affect the quality of brake operation in any way.

Small differences were also observed in the amount of heat conducted to the interior of the material. Material S1 demonstrated the worst insulation. At a depth of 3 mm from contact with the disk, it had the lowest temperature, amounting to about 414.5 K, which gives a difference of about 9.4 K in relation to the contact surface. This material best conducts heat energy into the interior due to conduction.

In the case of material S4, the temperature at the same depth, i.e., 3 mm, was close to 416.7 K, which is over 2 K higher than in S1. In relation to the temperature recorded at the contact surface, the difference is 8.7 K.

**Figure 6:**
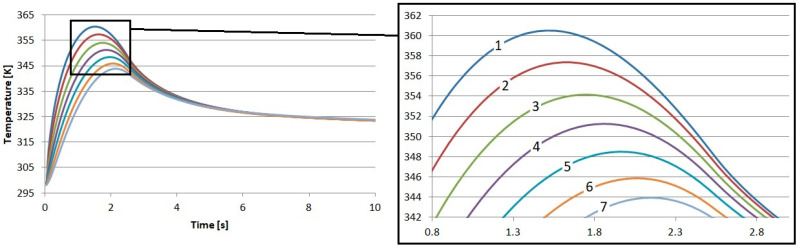
Results of numerical calculations of the brake disk cooperating with material S1: 1—depth 0.05 mm, 2—depth 0.5 mm, 3—depth 1 mm, 4—depth 1.5 mm, 5—depth 2 mm, 6—depth 2.5 mm, and 7—depth 3 mm.

**Figure 7:**
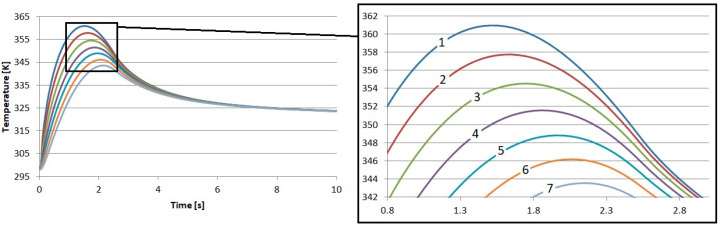
Results of numerical calculations of the brake disk cooperating with material S2: 1—depth 0.05 mm, 2—depth 0.5 mm, 3—depth 1 mm, 4—depth 1.5 mm, 5—depth 2 mm, 6—depth 2.5 mm, and 7—depth 3 mm.

**Figure 8:**
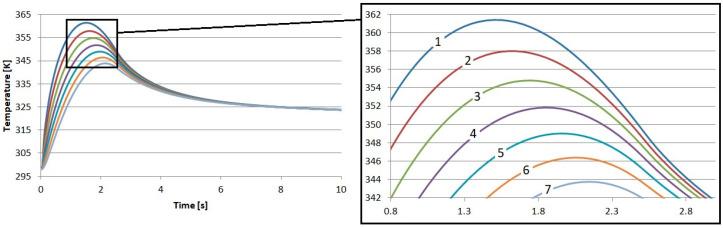
Results of numerical calculations of the brake disk cooperating with material S3: 1—depth 0.05 mm, 2—depth 0.5 mm, 3—depth 1 mm, 4—depth 1.5 mm, 5—depth 2 mm, 6—depth 2.5 mm, and 7—depth 3 mm.

**Figure 9:**
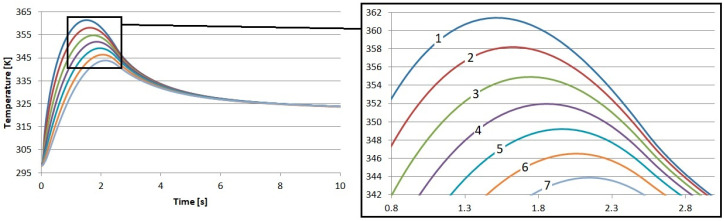
Results of numerical calculations of the brake disk cooperating with material S4: 1—depth 0.05 mm, 2—depth 0.5 mm, 3—depth 1 mm, 4—depth 1.5 mm, 5—depth 2 mm, 6—depth 2.5 mm, and 7—depth 3 mm.

As a result of our tests, the temperature in the brake disk was also recorded. The measurement points were placed exactly the same as they were in the pad. The first point was placed 0.05 mm below the surface. The second was placed at a depth of 0.5 mm, and we continued to place points every 0.5 mm up to a depth of 3 mm. The temperature profiles obtained in this way are presented in Figure 6, Figure 7, Figure 8 and Figure 9.

The influence of the previously described differences in the thermal and physical properties of the proposed friction materials is also noticeable in the recorded temperature profiles of the brake disks. It can be seen that the highest temperature near the surface of the brake disk occurred when working with the material reinforced with flax fibers. The highest temperature was 361.4 K. This is a value that is 0.9 K higher than that for the S1 material. This means that during the cooperation of the cast iron disk with the pad made of S1, the disk received less heat.

It is shown here that there is a certain advantage of using flax fibers for reinforcement. They allow for better heat dissipation from the pad to the disk, which, thanks to the fact that it has ventilation channels and performs a rotary movement, releases heat to the atmosphere in a much better manner. This is important because it accelerates the cooling of the brake pad and, thus, reduces the risk of the extremely dangerous phenomenon of fading.

## 4. Conclusions

This paper proposes an innovative composition of a composite friction material for passenger car brakes. The innovation consisted in replacing conventional aramid reinforcements with flax fibers. This change is significant because it allows for the elimination of one of the brake pad components, the production of which involves the use of toxic chemical compounds. Flax is a much more ecological solution. It allows not only for a production process that is cleaner from an ecological point of view but also for reducing the emission of harmful compounds as wear products.

In our previous paper [39], the impact of the proposed change on tribological parameters was determined. It turned out that the change in the reinforcement method was not found to have a significantly negative effect on the value of the friction coefficient or the abrasive wear rate coefficient. In this paper, an attempt was made to estimate how the change in reinforcement would affect the course of the heating process. The following was determined:

- There is a small difference in the maximum temperature values reached by the S1–S4 materials.
- The highest temperature, which was 425.3 K, was reached by material S4.
- The S1 material achieved the lowest heat value, registering a value of 423.9 K.
- Material S4, i.e., the one in which aramid was completely replaced with flax fibers, showed the best thermal insulation; most heat was therefore transferred to the shield, which facilitates cooling and reduces the risk of fading.

## Figures and Tables

**Table 1:** Compositions of individual groups of samples.

Component	% of Total Weight			
	S1	S2	S3	S4
Brass (CuZn20)	12	12	12	12
Cooper (Cu)	25	25	25	25
Steel (0.18% C, 0.5% Si, 1.65% Mn, 0.05% P, 0.02% S, 0.08% Mo)	7	7	7	7
Aramid	12	8	4	0
Flax fibers	0	4	8	12
Resin	17	17	17	17
Graphite (C)	5	5	5	5
Fly ash	18	18	18	18
Cast iron EN-GJS-400-12	4	4	4	4

**Table 2:** Physical and thermal data of friction materials.

Parameter	S1	S2	S3	S4	Disk
Thermal conductivity [W/m·K]	120	122	118	119	45
Density [kg/m^3^]	2980	2960	2975	2980	7850
Heat capacity at constant pressure [J/kg·K]	1254	1215	1186	1163	460

## Data Availability

Data are contained within the article.
